# Oral Hygiene Management of Hematologic Patients Undergoing Auto-Transplantation: An Observational Study

**DOI:** 10.3390/dj13030115

**Published:** 2025-03-05

**Authors:** Marco Miceli, Elisabetta Carli, Enrico Orciolo, Maria Rita Giuca, Rossana Izzetti

**Affiliations:** 1 Unit of Dentistry and Oral Surgery, Department of Surgical, Medical and Molecular Pathology and Critical Care Medicine, University of Pisa, 56126 Pisa, Italy; 2 Unit of Hematology, University Hospital of Pisa, 56126 Pisa, Italy

**Keywords:** dental prophylaxis, hematopoietic stem cell transplantation, motivation, oral hygiene

## Abstract

**Background/Objectives:** The present study evaluated the effectiveness of a prevention protocol involving oral hygiene, instructions, and motivation in patients eligible for hematopoietic stem cell transplantation (HSCT). **Methods:** Consecutive patients scheduled for HSCT were enrolled. All patients received a complete dental examination aimed at assessing periodontal health status through periodontal charting and registration of the levels of plaque and inflammation. Subsequently, patients received professional oral hygiene care and were instructed on and motivated toward oral prophylaxis prior to HSCT. Upon engraftment, patients received dedicated instructions to improve oral hygiene maneuvers. After HSCT, patients were re-evaluated for periodontal clinical parameters. The Oral Health Impact Profile-14 (OHIP-14) questionnaire was also administered to evaluate perceived oral health status. **Results:** Thirty-five patients (18 males and 17 females, with a mean age of 43.28 ± 11.73 years) were enrolled. An overall improvement was noted in periodontal parameters. However, despite plaque reduction, inflammation was still present because of the treatment. The OHIP-14 score slightly worsened after HSCT. **Conclusions:** Professional oral hygiene and prophylaxis proved effective in improving the oral health status of patients undergoing HSCT, potentially impacting post-treatment outcomes. Further assessment is needed to validate these results.

## 1. Introduction

Oral hygiene management is a critical component in the care of patients undergoing hematopoietic stem cell transplantation (HSCT) for hematologic diseases [1]. This patient population is particularly vulnerable to oral complications due to the intense chemotherapy and immunosuppressive regimens associated with the transplantation process as the preconditioning regimens for transplantation can cause direct damage to the oral mucosa [2,3,4].

Mucositis is a common and often severe complication following HSCT, characterized by inflammation, ulceration, and pain in the oral mucosa [5]. It typically develops as a result of conditioning regimens, including chemotherapy and/or radiation therapy, which lead to mucosal barrier disruption and increased susceptibility to infections [5]. Clinically, mucositis presents with erythema, edema, and painful ulcerative lesions, significantly impacting oral function, nutrition, and overall quality of life [5,6]. The severity of mucositis can vary, often peaking around 7–14 days post-HSCT, with recovery occurring as neutrophil counts begin to normalize. This condition not only causes significant discomfort but also serves as a potential entry point for systemic infections, which can be life-threatening in immunocompromised patients [6]. Effective management strategies, including pain control, oral hygiene protocols, and supportive care, are crucial in minimizing its impact and preventing secondary complications.

The importance of oral hygiene in this context cannot be underestimated. Poor oral health prior to transplantation can exacerbate the severity of mucositis and increase the risk of systemic infections [7]. Therefore, a comprehensive oral hygiene protocol is essential. This typically includes pre-transplant dental assessments, prophylactic dental treatments, and rigorous oral care regimens during and after the transplantation process [7].

Pre-transplant dental assessments are vital for identifying and treating existing oral conditions that could complicate the transplantation process [8]. This includes managing caries, periodontal disease, and other potential sources of infection. Early dental intervention can help mitigate the risk of oral and systemic complications during the period of immunosuppression [9].

During the transplantation process, maintaining oral hygiene is paramount. Patients are often advised to follow meticulous oral care routines, including the use of non-alcoholic mouthwashes, frequent brushing with a soft-bristled toothbrush, and regular interdental hygiene. The use of antiseptic mouthwashes containing chlorhexidine can help reduce microbial load in the oral cavity, thereby decreasing the risk of infections. However, care must be taken to balance antimicrobial efficacy with the potential for mucosal irritation [10].

The treatment of oral complications after HSCT focuses on prevention, symptom management, and infection control. Oral mucositis can be managed with cryotherapy, topical anesthetics, palifermin, and low-level laser therapy [11]. These interventions aim to reduce the severity and duration of mucositis, thus improving patient comfort and reducing the risk of secondary infections [11].

Maintaining oral hygiene through professional cleanings, chlorhexidine rinses, and soft toothbrushes is essential to prevent periodontal issues. Post-transplantation, ongoing oral care remains important as patients recover and their immune system gradually returns to normal function [12]. Regular dental check-ups and continued adherence to good oral hygiene practices help prevent long-term complications such as chronic graft-versus-host disease, which can have significant oral manifestations [13]. Additionally, patients should be educated about the importance of maintaining oral health and promptly addressing any new oral symptoms that may arise during the recovery phase [12,13].

The aim of the present study was to assess the effectiveness of a prevention program in patients scheduled for HSCT to evaluate the effects of a dedicated oral hygiene regimen on oral health status.

## 2. Materials and Methods

### 2.1. Study Design and Population

This study was a monocentric, non-randomized clinical trial conducted over 18 months, focusing on patients undergoing autologous hematopoietic stem cell transplantation (HSCT) for hematologic diseases. The study included patients treated at the Hematology Unit of Pisa University Hospital between March 2021 and December 2023. Participants were selected based on their eligibility for HSCT and their consent to participate in the study through convenience sampling. The study was approved by the IRB of the University Hospital of Pisa (protocol no. 15078) and registered in a clinical trials database (clinicaltrials.gov, NCT06755333). The study was conducted according to the principles outlined in the Declaration of Helsinki. All patients signed an informed consent form to be included in the study.

### 2.2. Eligibility Criteria

Patients were included based on the presence of the following characteristics: (i) patients aged 18–75 years; (ii) patients diagnosed with any hematopoietic disease (acute myeloid leukemia, chronic myelogenous leukemia, Non-Hodgkin lymphoma, myelodysplastic syndrome, acute lymphocytic leukemia, and multiple myeloma) requiring treatment with HSCT; (iii) acceptance of study participation; (iv) compliance to follow-up. Patients were excluded in cases of denial of consent to participate in the study.

### 2.3. Clinical Assessment

Clinical evaluation was performed at the Unit of Dentistry and Oral Surgery at the University Hospital of Pisa. A comprehensive dental examination was conducted by three examiners (MM, EC, and RI) following rigorous training and calibration sessions prior to the study beginning, ensuring an inter-examiner agreement exceeding 0.8.

The following parameters were assessed using a 3 instrument dental kit (1 mouth mirror, 1 straight probe, and 1 periodontal probe):

- Probing pocket depth (PPD) was measured as the distance from the free gingival margin to the bottom of the pocket by inserting a periodontal probe parallel to the longitudinal axis of the tooth (UNC 15, Hu-Friedy, Chicago, IL, USA) with a calibrated pressure of 0.3 N. All measurements were rounded to the nearest millimeter. Periodontal diagnosis was performed according to the 2017 classification [14].
- Full-Mouth Bleeding Score (FMBS) was measured dichotomously after periodontal probing. The mean bleeding score was indicated as the percentage of sites detected positive for bleeding on the total number of sites [15]. The Full-Mouth Plaque Score (FMPS) assessed the presence or absence of plaque on each tooth surface. The presence of plaque was evaluated as 1, while the absence was evaluated as 0, on 6 sites per tooth (distobuccal, buccal, mesiobuccal, distolingual, lingual, and mesiolingual). The mean plaque score was indicated as the percentage of sites found positive for the presence of plaque on the total number of sites [16]. The Calculus Index was measured using a probe and considering supragingival and subgingival calculus separately, with a score of 0 assigned in the absence of calculus and score of 3 assigned to supragingival calculus exceeding two-thirds of the crown and/or continuous bands of subgingival calculus [17].

The number of teeth and the presence of prosthetic rehabilitations were also registered. Patients were administered the Oral Health Impact Profile-14 (OHIP-14) questionnaire to evaluate the social impact of oral disorders on an individual’s well-being. It comprises 14 questions across seven domains: functional limitation, physical pain, psychological discomfort, physical disability, psychological disability, social disability, and handicap. Responses are scored on a 5-point Likert scale (0–4), with total scores ranging from 0 to 56, where higher scores indicate a greater negative impact on quality of life. Scores are categorized as minimal (0–14) or increasing in severity beyond this range [18].

After HSCT, the presence of mucositis and dysphagia were investigated, being common consequences of HSCT [19]. Mucositis was classified according to the Oral Mucositis Assessment Scale (OMAS) established by Sonis et al. [20]. The OMAS is an objective and site-specific tool used to assess the severity of oral mucositis by evaluating erythema and ulceration across nine intraoral sites, including the upper and lower lips, right and left buccal mucosa, right and left lateral tongue, ventral tongue/floor of mouth, soft palate/fauces, and hard palate. Each site is scored separately for erythema on a scale from 0 to 2, where 0 represents no erythema, 1 indicates mild erythema, and 2 signifies severe erythema, while ulceration is scored from 0 to 3, with 0 indicating no ulcers, 1 for ulceration smaller than 1 cm^2^, 2 for ulceration between 1 and 3 cm^2^, and 3 for ulceration larger than 3 cm^2^. The final OMAS score is calculated as the average of all site scores, making it a continuous rather than categorical scale.

Dysphagia was categorized according to the Italian version of the Functional Oral Intake Scale (FOIS) [21]. Scores range between 1 and 7, with 1 indicating an absence of oral intake, 2–3 tube-dependence with some oral intake, 4–5 total oral intake with restrictions, and 6–7 normal oral intake.

### 2.4. Study Timepoints

The patients were assessed individually at three timepoints:

- T0 (baseline): initial professional oral hygiene session before chemotherapy and implementation of the pre-transplant oral hygiene protocol, and OHIP-14 administration.
- T1 (day +1 post reinfusion): switch to the post-transplant oral hygiene protocol.
- T2 (upon engraftment): final clinical assessment and OHIP-14 administration.

At baseline (T0), all patients underwent a professional oral hygiene session before starting chemotherapy as part of the routine pre-transplant procedure. During this session, periodontal charting was performed. Patients then received oral hygiene instructions including information on the brushing technique to be used, the performance of interdental hygiene, the importance of chemical agents, and motivation toward oral prophylaxis.

The use of the following devices and products was recommended:

- A manual super soft toothbrush (Curaprox 3960, Curaden AG, Kriens, Switzerland) to be used according to the modified Bass technique.
- An antibacterial toothpaste containing natural enzymes (lactoperoxidase, glucose oxidase, and lysozyme), lactoferrin, extracted colostrum, whey protein, moisturizers, and xylitol (Bioxtra, BiopHarm Srl, Milan, Italy).
- An alcohol-free mouthwash containing lactoferrin, lactoperoxidase, glucose oxidase, potassium thiocyanate, and whey protein (Oralis) to be used twice daily.

Upon admission, patients received pre-transplant conditioning chemotherapy. At T1 (first day after transplantation), motivation and oral hygiene instructions were repeated to the patients. The patients were advised to use the following devices and products:

- A manual ultra soft toothbrush (Curaprox 5460, Curaden AG, Kriens, Switzerland).
- An antibacterial toothpaste containing natural enzymes (lactoperoxidase, glucose oxidase, and lysozyme), lactoferrin, extracted colostrum, whey protein, moisturizers, and xylitol (Bioxtra, BiopHarm Srl, Milan, Italy), which is a mouthwash containing polyvinylpyrrolidone, maltodextrin, propylene glycol, hydroxyethylcellulose, and Verbascoside (Syringa vulgaris extract) (Mucosyte, BiopHarm Srl, Milan, Italy) to be used three times daily to alleviate pain and inflammation.

### 2.5. Sample Size Estimation

Sample size estimation was performed using a study by Gennai et al. [22] as the reference, with a power of 95% and α = 0.05. This study reported reference proportions for FMPS reduction of 22% for the combination of a toothbrush with interdental brushes. Assuming that the study group receiving a toothbrush and interdental brushes and oral hygiene instructions would show an approximately 15% difference, a minimum of 30 patients would be required to achieve 95% power. To compensate for potential dropouts, a total of 35 patients were included in the study.

### 2.6. Statistical Analysis

All analyses were conducted using Jamovi software (The Jamovi project 2024, Version 2.5, retrieved from https://www.jamovi.org, accessed on 9 May 2024). The normality of data distribution was assessed using a Shapiro–Wilk test. The statistical analysis involved descriptive statistics to summarize patient demographics and baseline characteristics, and inferential statistics using paired *t*-tests or non-parametric equivalents to compare clinical parameters before and after the intervention. *p*-value was set at *p* < 0.05.

## 3. Results

Thirty-five patients (18 males and 17 females) were enrolled. The mean age was 43.3 years (SD 11.7). All patients completed the study. In Table 1, demographic characteristics of the study sample are described. No significant differences were present at baseline between males and females in terms of age and hematological disease distribution. The most frequently encountered oral condition was plaque-induced gingivitis. Only three patients were diagnosed with periodontitis (one at stage I and two at stage II [14]). Males and females were comparable in terms of oral health parameters at baseline.

Following HSCT (Table 2), the mean duration of neutropenia was 12.1 days. Mucositis was detected for a week post-HSCT on average, with mild to severe erythema and the presence of ulcerations > 1 cm^2^ in 77.1% (27 patients) and 68.6% (24 patients) of cases, respectively.

At T2, clinical parameters were re-assessed. Despite undergoing HSCT, there was an overall improvement in clinical parameters. Female patients showed slightly better oral health parameters than males, but the difference was not statistically significant. A significant improvement was noted between pre- and post-HSCT in terms of PPD values (*p* < 0.05), FMPS (*p* < 0.001), and CI (*p* < 0.05). Although FMBS values were observed to have decreased between the two timepoints, such variation did not reach statistical significance. OHIP-14 scores were observed to have slightly worsened, but the differences were not significant. (Table 3).

## 4. Discussion

The present results highlight that oral hygiene performance, along with dedicated instructions and motivation, could improve the oral health status of patients undergoing HSCT. Importantly, although a mild worsening of OHIP-14 scores was noted following HSCT and the development of mucositis, such a modification was not statistically significant. This finding potentially suggests that both the prophylaxis performed prior to HSCT and the oral hygiene protocol adopted could be beneficial in attenuating symptoms related to post-HSCT mucositis. These results align with findings from other studies, emphasizing the role of preemptive oral care in mitigating post-transplant complications [23,24,25,26].

The general oral health of patients undergoing HSCT is often inadequate. Fernandes et al. [23] reported that candidates for HSCT had a high prevalence of oral pathological conditions, including visible plaque, gingival inflammation, and a widespread need for dental treatment, with nearly 98% requiring intervention before transplantation. However, less than half of these patients had visited a dentist within six months prior to HSCT, highlighting a significant gap in pre-transplant oral care. Uutela et al. [24] examined the oral health of HSCT recipients before transplantation, comparing them to healthy age- and sex-matched controls. Findings revealed that HSCT recipients had significantly lower stimulated saliva flow rates, a higher DMFT (decayed, missing, and filled teeth) index, and more caries lesions than controls, indicating poorer oral health. However, no significant differences in periodontitis prevalence were observed. The authors highlighted the importance of preventive dental strategies before HSCT to mitigate oral complications and improve patient outcomes. More recently, Sabancı and Kuku [25] investigated the relationship between oral health status and infectious complications in HSCT recipients during the neutropenic phase. Patients with higher DMFT scores and more sites with a probing depth ≥ 4 mm were more likely to develop febrile neutropenia, suggesting that poor dental health could contribute to febrile neutropenia, highlighting the importance of pre-transplant oral care.

It is estimated that nearly 60% of patients preparing for HSCT experience oral symptoms and dental diseases [26]. Despite variations in oral hygiene practices, professional dental care and oral hygiene instructions may reduce post-HSCT complications, highlighting the need for standardized dental screening before HSCT to mitigate potential risks and improve overall patient outcomes [26].

In our sample, at baseline, patients presented with inflammation and plaque accumulation. Importantly, the performance of professional oral hygiene along with repeated oral hygiene instructions concurred with an improvement in periodontal parameters, consistent with the literature [27]. Following the follow-up period, significant improvements were observed in key clinical parameters of periodontal status, particularly PPD, FMPS, and CI. The significant reduction in PPD values indicates a measurable improvement in periodontal health, suggesting reduced inflammation and better overall tissue stability. Similarly, the highly significant decrease in FMPS reflects a substantial reduction in plaque accumulation, which is a critical factor in periodontal disease progression and treatment success. Improvement in the CI further supports these findings, indicating a lower presence of calculus deposits. These statistically significant changes in periodontal health may have a positive impact on post-HSCT recovery, likely due to improved oral hygiene measures.

Chemotherapy, conditioning regimens, and prior medications further predispose patients to infections and long-term oral complications, which can significantly impact their quality of life [26]. Oral complications in HSCT recipients significantly impact morbidity and, in some cases, mortality [28]. While conditioning regimens influence the risk of oral mucositis, individual susceptibility varies, with genetic predispositions playing a role. Conditions like oral mucositis, hyposalivation, taste alterations, and oral infections appear interrelated, potentially linking oral and systemic complications, including bacteremia and sepsis. Pre-existing inflammation, such as periodontitis, may amplify HSCT-related inflammatory responses, exacerbating complications [28]. Elad et al. [29] emphasized that oral complications, including mucositis, xerostomia, and candidiasis, occur frequently in HSCT patients, and preventive measures, such as fluconazole prophylaxis and regular oral hygiene protocols, are crucial for reducing the prevalence of these issues.

Oral mucositis remains a major complication, with incidence rates between 73 and 86% [30]. A link between oral mucositis development and total body irradiation, body mass index, and genetic factors with oral mucositis risk, has been hypothesized. Evidence suggests that pre-transplant oral care protocols reduce oral mucositis incidence, yet preventive measures such as cryotherapy and palifermin are not universally adopted [30]. Multimodal approaches are recommended, including oral care, antiseptic mouthwashes, and photo-biomodulation therapy. In the literature, various agents are reported, including sucralfate, curcumin, Lactobacillus-based lozenges, grape seed extract, and benzydamine [31]. However, to date, there is a lack of a gold standard for preventing and treating oral mucositis [31]. Despite the well-documented importance of oral health in HSCT patients, many do not receive specialist dental examinations before transplantation. Reports indicate that only 40% of patients undergo such evaluations, leaving the majority at risk of preventable complications [32]. A lack of standardized, evidence-based clinical guidelines for dental management in adult HSCT patients contributes to this issue, resulting in inconsistencies among transplant centers. Opinions differ on the necessity of eliminating potential infection foci in the oral cavity prior to conditioning therapy. Some experts advocate for comprehensive oral sanitation, while others recommend a more conservative approach, balancing the risks of invasive procedures, such as infection and prolonged bleeding, with the potential for delaying cancer treatment [32,33]. Evidence suggests that eliminating potential infective foci can significantly reduce complications related to immunosuppression, but this must be carefully weighed against procedural risks [33]. Epstein et al. [32] highlight that oral mucositis, as one of the most debilitating side effects of HSCT, could be mitigated by well-structured dental management protocols and the timely use of supportive care measures, such as cryotherapy and keratinocyte growth factors.

Furthermore, patients are often insufficiently informed about the adverse effects of HSCT on the oral cavity. Less than half of HSCT patients are aware of potential oral complications, such as mucositis, infections, and salivary gland dysfunction, and those who receive information often find it insufficiently detailed to enable effective oral health management [34]. This knowledge gap underscores the importance of improving patient education and interdisciplinary communication among oncologists, dentists, and patients. Proper education on oral hygiene practices, including motivation to adopt a robust hygiene regimen weeks before transplantation, can play a pivotal role in reducing oral complications. Regular dental check-ups, particularly in the first-year post-transplant, are also highly recommended [34]. Comprehensive care plans should include patient education to address complications like dry mouth, oral pain, and mucositis, improving both short- and long-term outcomes for HSCT recipients [35].

Prior to undergoing HSCT, patients often present with various oral health issues, including periodontal diseases, dental caries, and mucosal lesions. These conditions can serve as potential sources of infection during the immunocompromised state post-transplantation, leading to increased morbidity. As observed in our sample, baseline characteristics included inflammation and plaque accumulation, both of which are key contributors to the development of infectious complications. From this perspective, the primary goal of pre-HSCT dental management is to identify and address these oral health problems to minimize the risk of infections originating from the oral cavity during the acute phase following HSCT. This involves thorough dental evaluations and necessary treatments to eliminate potential infection foci [36].

Protecting oral tissues during HSCT is crucial due to the high incidence of oral complications, such as mucositis, which affects about 80% of patients undergoing this therapy. The compromised oral barrier function in immunosuppressed patients allows for the translocation of microorganisms, increasing the risk of systemic infections. Therefore, maintaining optimal oral hygiene and health is essential to preserve the integrity of oral tissues and prevent such complications [37]. Enhanced periodontal management before HSCT can lead to additional positive outcomes. By reducing oral inflammation and infection, patients may experience a decrease in the severity and duration of oral mucositis, leading to improved comfort and quality of life during treatment. Furthermore, effective oral care can prevent delays or modifications in HSCT protocols due to oral health issues, thereby contributing to better overall treatment outcomes [38].

The present study has limitations that must be considered. The lack of a control group, the small sample size, and the absence of randomization hinder the ability to draw firm conclusions. Moreover, the inclusion of patients through convenience sampling may have increased the risk of selection bias. Despite these limitations, the results provide valuable insights into the role of a dedicated oral hygiene regimen prior to HSCT. They further emphasize the unmet need for structured oral hygiene training and motivation in this patient population. Future studies with larger cohorts, control groups, and randomization are needed to strengthen these findings and develop comprehensive care protocols.

In conclusion, oral health care is a critical aspect of patient management in HSCT, yet it remains an under-addressed area. Indeed, providing professional oral hygiene care and tailored oral hygiene instructions may have clinical relevance in improving periodontal parameters, particularly in HSCT patients, by reducing inflammation, plaque accumulation, and overall periodontal disease progression. To improve outcomes, consistent and comprehensive dental management protocols must be established, incorporating increased patient education, awareness campaigns, and regular dental follow-ups. Developing standardized guidelines and conducting further research will be essential to optimize care and reduce the burden of oral complications in HSCT patients.

## Figures and Tables

**Table 1:** Baseline characteristics of the study sample.

Baseline Characteristics	
Males	18 (51.4%)
Females	17 (48.6%)
Age	43.3 (±11.7)
Platelet count < 50,000 K/uL	11 (31.4%)
Number of teeth	26 (±4.7)
Presence of plaque-induced gingivitis	26 (74.3%)
Presence of periodontitis	3 (8.6%)
Presence of prosthetic rehabilitation	30 (85.7%)
Fixed	26 (74.3%)
Removable	4 (11.4%)
Hematologic disease	
Acute myeloid leukemia	8 (23%)
Chronic myelogenous leukemia	5 (14%)
Non-Hodgkin lymphoma	7 (21%)
Myelodysplastic syndrome	5 (14%)
Acute lymphocytic leukemia	5 (14%)
Multiple myeloma	5 (14%)

**Table 2:** Post-HSCT symptom development and timing.

Post-Hematopoietic Stem Cell Transplantation Symptoms	Total
Duration of neutropenia (days)	12.1 (±3.0)
Duration of fever (days)	4.0 (±1.8)
Duration of mucositis (days)	7.1 (±1.9)
OMAS score erythema	1.5 (±0.3)
OMAS score ulceration	2.7 (±1.8)
Presence of dysphagia	13 (37%)
FOIS score	3.4 (±1.1)

Abbreviations: FOIS: Functional Oral Intake Scale; OMAS: Oral Mucositis Assessment Scale.

**Table 3:** Oral health status and oral health-related quality of life at baseline and T2 follow-up.

	T0	T2	*p*-Value
PPD	3.9 mm (±1.2 mm)	3.3 mm (±0.3 mm)	0.038 *
FMBS	35.41% (±5.18%)	31.51% (±6.84%)	0.190
FMPS	29.90% (±8.23%)	16.19% (±7.03%)	0.0007 **
CI	2.15 (±0.62)	1.4 (±0.5)	0.044 *
OHIP-14	16.51 (±4.43)	17.29 (±4.22)	0.298

Abbreviations: CI: Calculus Index; FMBS: Full-Mouth Bleeding Score; FMPS: Full-Mouth Plaque Score; OHIP-14: Oral Health Impact Profile 14; PPD: Probing Pocket Depth; * *p* < 0.05; ** *p* < 0.01.

## Data Availability

The original contributions presented in this study are included in the article. Further inquiries can be directed to the corresponding author.
